# Navigating the Therapeutic Landscape of Non-Infectious Uveitis Despite Major Gaps

**DOI:** 10.3390/biomedicines13112772

**Published:** 2025-11-13

**Authors:** Uwe Pleyer, Lynn S. zur Bonsen

**Affiliations:** 1 Department of Ophthalmology, Charité—Universitätsmedizin Berlin, Campus Virchow-Klinikum, Augustenburger Platz 1, 13353 Berlin, Germany; 2 Department of Ophthalmology, Charité—Universitätsmedizin Berlin, Corporate Member of Freie Universität Berlin and Humboldt—Universität zu Berlin, Augustenburger Platz 1, 13353 Berlin, Germany

This Special Issue of *Biomedicines*, on “Recent Advances in Diagnosis and Therapeutic Strategies in Intraocular Inflammation”, reflects both the opportunities and the enduring limitations of current therapies. Although progress has been made in the management of non-infectious uveitis (NIU), the field still lags behind other comparable areas of medicine.

A clear trend has emerged toward the use of local treatments, such as long-acting corticosteroid implants, which provide extended therapeutic benefit. At the same time, however, both the use and development of targeted biomodulators—including biologic DMARDs or small-molecule agents—remain limited. In fact, treatment for NIU continues to rely primarily on corticosteroids, conventional immunosuppressants, and a single bDMARD, with no approved small-molecule therapies available to date.

When viewed in the broader landscape of autoimmune diseases, the therapeutic pipeline for NIU appears strikingly sparse compared with conditions such as psoriasis, rheumatoid arthritis, or multiple sclerosis [1,2,3,4]. In these fields, the past two decades have witnessed an explosion of novel biologics, targeted small molecules, and oral agents that have redefined the standards of care.

This discrepancy does not merely reflect scientific “neglect” but rather the unique challenges inherent to uveitis as a broad disease spectrum. Unlike psoriasis, which manifests with relatively uniform cutaneous lesions, or multiple sclerosis, which has well-defined diagnostic criteria and validated endpoints such as MRI lesion load, uveitis is a heterogeneous condition. It encompasses anterior, intermediate, posterior, and panuveitis form, each with diverse etiologies ranging from idiopathic to systemic autoimmune associations.

The absence of a single, unifying pathophysiological pathway complicates the development of targeted therapies that can be broadly applied across the disease spectrum. Clinical trial design is further hampered by the lack of universally accepted endpoints. Whereas dermatologists can rely on PASI scores and neurologists on relapse rates or disability scales, ophthalmologists struggle with surrogate markers that vary widely, such as reduction in vitreous haze, improvement of best-corrected visual acuity or central retinal thickness (CRT), resolution of macular edema, or prevention of recurrence. These endpoints are not only heterogeneous but also influenced by prior (macular) damage and subjective grading. Consequently, regulatory agencies have been cautious in approving new agents, demanding robust and reproducible outcomes.

Ethnic and geographic variability further adds to the complexity of NIU. Diseases such as Behçet’s uveitis are more prevalent in specific regions, while birdshot chorioretinopathy predominantly affects Caucasians. This global heterogeneity makes it challenging to recruit large and representative cohorts for randomized controlled trials.

A probably underestimated factor is the relatively low profile of patient advocacy groups in ophthalmology in general and in uveitis in particular. In dermatology and neurology, strong patient organizations and lobbying efforts have successfully raised awareness. Active lobbying likely influenced funding priorities and attracted pharmaceutical investment. By contrast, uveitis remains a niche condition in the public’s “eye”, with limited advocacy and little political attention. Pharmaceutical companies, understandably driven by market size and return on investment, are less motivated to invest in drug development for a disease that is both rare and fragmented. The result is stagnation in targeted drug innovation.

Returning to the topic of recent advances in therapeutic strategies for NIU, the overarching therapeutic objectives appear straightforward: to suppress ocular inflammation, prevent recurrence, and preserve vision. In practice, however, the path to achieving these goals is considerably more complex.

As already noted, the fundamental therapeutic options can broadly be divided into local and systemic approaches. Local treatment is particularly appealing when long-term efficacy can be achieved. A recent meta-analysis by Yeo et al. confirmed that the 0.19 mg fluocinolone acetonide implant provides long-term disease control, in many cases over several years [5]. Importantly, it is highly effective in the management of cystoid macular edema (CME), frequently with superior outcomes compared to bDMARDs. Moreover, implants have proven effective across a range of indications. Importantly, success is independent of patient adherence. It is therefore unsurprising that a broad European consensus has led to a proposed stepwise “traffic light” treatment strategy for NIU management [6]. On the other hand, as might be expected, local secondary adverse effects—particularly elevated intraocular pressure and cataract formation—are virtually unavoidable and, with repeated use, almost obligatory. While patients experience mental relief from fewer injections, anxiety about permanent implants and side effects persists. Financial strain is considerable: implants are expensive, and their cost-effectiveness remains debated, being favorable in unilateral but not in bilateral uveitis. Surgical risks, though low, still concern many patients.

Local therapy is thus most suitable for unilateral, isolated ocular involvement, particularly in pseudophakic patients. Systemic therapy, by contrast, is preferred in patients with underlying systemic diseases.

The advent of Adalimumab, the first and only EMA- and FDA-approved bDMARD for NIU, marked a paradigm shift [7,8]. Other biologics such as infliximab, tocilizumab, and interferons are used off label. These agents are highly effective in refractory uveitis, particularly in posterior and panuveitis, and have clear steroid-sparing potential. Subcutaneous administration allows home use, and the relatively long dosing intervals further enhance convenience. However, Adalimumab carries risks, including serious infections (e.g., tuberculosis, opportunistic infections) and rare but potentially serious demyelinating disorders. The development of anti-drug antibodies (ADA), which can markedly reduce efficacy, is another yet-underestimated issue. The immunogenicity of adalimumab has been a recurring topic across immune-mediated diseases, and the recent uveitis study by zur Bonsen et al. contributes an important ophthalmic perspective to this broader narrative [9]. These findings—an inverse correlation between ADA levels and drug concentration, earlier detection of high-titer antibodies, and the potential benefit of routine monitoring—echo established observations from other specialties [10,11,12,13,14].

In gastroenterology, particularly in Crohn’s disease and ulcerative colitis, ADA formation is a well-recognized mechanism underlying secondary loss of response. Up to 40% of patients develop ADAs within the first year of anti-TNF therapy, and proactive therapeutic drug monitoring (TDM) has become standard practice in many centers [10,11]. Combination therapy with immunomodulators such as methotrexate is routinely employed to mitigate immunogenicity and prolong drug survival.

In rheumatology, similar patterns are observed, with ADA positivity predicting lower trough levels, diminished clinical response, and earlier treatment discontinuation [12]. Here, too, methotrexate co-medication appears protective, and TDM is increasingly integrated into practice, albeit often in a reactive rather than proactive manner.

In dermatology, the picture is more complex. Although ADA prevalence can be high in psoriasis cohorts, its correlation with clinical outcomes is inconsistent [13]. This has led to skepticism regarding the utility of routine antibody testing, with treatment decisions still largely guided by clinical response rather than laboratory monitoring.

Placed in this context, the uveitis data appear more aligned with gastroenterology and rheumatology than with those in dermatology. The stakes in ophthalmology are unfortunately high: delayed recognition of treatment failure risks irreversible vision loss. The observation that nearly half of ADA-positive uveitis cases were identified through routine testing—frequently prior to the onset of clinical symptoms—strengthens the argument for proactive monitoring in this field [9,14].

Taken together, the comparison of local and systemic therapies reveals a nuanced landscape. Local therapy is best suited for unilateral or purely ocular disease. Local therapies avoid systemic side effects but carry ocular risks and entail significant costs. Since all currently available implants are steroid-based, pseudophakic patients and steroid “non-responders” in terms of intraocular pressure are particularly suitable candidates for this approach. Systemic therapy is generally the first choice when uveitis is associated with systemic autoimmune conditions or in cases of bilateral disease. This approach often provides comprehensive disease control but comes at the cost of systemic toxicity, psychological burden, and high financial expense [15].

From the patient’s perspective, therapy extends far beyond medical calculations. It affects quality of life, mental resilience, and career and family planning as well as financial stability. Many patients oscillate between gratitude for preserved vision and frustration at the relentless treatment burden. The mental commitment—repeated injections, implant procedures, ongoing monitoring, side effects—is enormous. Added to this are the uncertainties of disease course, the fear of vision loss, and threats to independence, often affecting patients at a young age.

Therefore, the search for new, targeted, and safer therapies must continue. Future progress will rely on both scientific innovation and collaboration among clinicians, researchers, regulators, and patient advocates to close the remaining therapeutic gap in non-infectious uveitis.
